# The Role of Matrix Metalloproteinases in Hemorrhagic Transformation in the Treatment of Stroke with Tissue Plasminogen Activator

**DOI:** 10.3390/jpm13071175

**Published:** 2023-07-23

**Authors:** Valentina A. Babenko, Ksenia S. Fedulova, Denis N. Silachev, Parvaneh Rahimi-Moghaddam, Yulia N. Kalyuzhnaya, Svetlana V. Demyanenko, Egor Y. Plotnikov

**Affiliations:** 1 Belozersky Institute of Physico-Chemical Biology, Lomonosov Moscow State University, 119991 Moscow, Russia; babenkova@belozersky.msu.ru (V.A.B.); xenia.fedulova@yandex.ru (K.S.F.); 2 Department of Pharmacology, School of Medicine, Iran University of Medical Sciences, Tehran 14496-14535, Iran; rahimi.p@iums.ac.ir; 3 Academy of Biology and Biotechnology, Southern Federal University, 344090 Rostov-on-Don, Russia; ykalyuzhnaya@sfedu.ru (Y.N.K.); svdemyanenko@sfedu.ru (S.V.D.)

**Keywords:** ischemic stroke, thrombolytic therapy, tissue plasminogen activator, hemorrhagic transformation, matrix metalloproteinases

## Abstract

Ischemic stroke is a leading cause of disability and mortality worldwide. The only approved treatment for ischemic stroke is thrombolytic therapy with tissue plasminogen activator (tPA), though this approach often leads to a severe complication: hemorrhagic transformation (HT). The pathophysiology of HT in response to tPA is complex and not fully understood. However, numerous scientific findings suggest that the enzymatic activity and expression of matrix metalloproteinases (MMPs) in brain tissue play a crucial role. In this review article, we summarize the current knowledge of the functioning of various MMPs at different stages of ischemic stroke development and their association with HT. We also discuss the mechanisms that underlie the effect of tPA on MMPs as the main cause of the adverse effects of thrombolytic therapy. Finally, we describe recent research that aimed to develop new strategies to modulate MMP activity to improve the efficacy of thrombolytic therapy. The ultimate goal is to provide more targeted and personalized treatment options for patients with ischemic stroke to minimize complications and improve clinical outcomes.

## 1. Introduction

The American Stroke Association defines ischemic stroke as a neurological dysfunction characterized by the presence of a focal infarction in the central nervous system or retina [1]. This pathology is the second leading cause of death and the third leading cause of disability in the adult population worldwide [2]. Although ischemic stroke has several etiologies, the most common cause is a thrombotic or embolic incident of cerebral blood vessels, which leads to the formation of an ischemic focus, with blood circulation falling below a critical level and disrupting the function of nervous tissue. Irreversible damage to cells in the ischemic focus occurs within a few tens of minutes, while the peri-infarct region known as the penumbra forms, where blood flow is insufficient for normal cell function. However, due to collaterals and redistribution of blood through the Willis circuit, cells in the penumbra region can survive or fully recover their functions after blood flow is resumed [2,3].

Early treatment of patients with acute ischemic stroke is crucial, and recanalization therapy is one of the main strategies recommended in current treatment guidelines. Recanalization therapy involves extraction of blood clots (thrombectomy) and thrombolysis. Thrombolysis involves the administration of thrombolytic agents that induce lysis of the blood clot and restoration of cerebral blood flow, thereby restoring brain cell function. Tissue plasminogen activator (tPA, alteplase) is one such pharmacological agent used in thrombolytic therapy [4]. Currently, tPA is the main drug used to treat patients with acute ischemic stroke, and it is widely used in clinical practice with proven efficacy. However, treatment with tPA is associated with some serious side effects, especially hemorrhagic transformation. Hemorrhagic transformation may occur when intraparenchymal or intraventricular hemorrhage occurs in an ischemic brain area, which increases the patient’s risk of death [5]. Therefore, strategies to reduce hemorrhagic transformation are critical in terms of improving clinical outcomes in patients who receive thrombolytic therapy with tPA. One of the most important mechanisms of this phenomenon is a change in the activity of matrix metalloproteinases (MMPs), which can be modulated under the influence of tPA.

Therefore, to increase the efficacy of ischemic stroke therapy and decrease the likelihood of undesirable hemorrhagic transformations, it is greatly important to study the mechanisms of side effects during thrombolytic therapy with tPA and find ways to correct these issues.

## 2. Thrombolytic Therapy with Tissue Plasminogen Activator

It is worth noting that for a long time, there was no pathogenetic therapy for ischemic stroke. It was only after 1995 that the results of a National Institute of Neurological Diseases and Stroke (NINDS) study were published, and thrombolytic therapy then began to be used in clinical settings [3]. A randomized double-blind trial of the effect of tPA was conducted in 333 patients with ischemic stroke, demonstrating the clinical efficacy of this agent, which is mainly expressed via a reduction in disability compared to the control group members who received placebo [6]. Currently, tPA is the only agent approved by the US Food and Drug Administration (FDA) for thrombolytic treatment of ischemic stroke [7].

tPA is a glycoprotein with a molecular weight of approximately 70 kDa, and it belongs to the serine protease family. Endothelial cells secrete it as a single-stranded polypeptide (sc-tPA). After cleavage by trypsin or plasmin, it assumes a catalytically active form and becomes a double-stranded polypeptide (tc-tPA). It consists of light and heavy chains with lengths of 275–276 residues and forms a functional complex [8]. The main mechanism of action of tPA is based on its protease activity, and it is able to initiate the dissolution of the fibrin clot. tPA triggers the mechanism of fibrinolysis by binding to the fibrin of the thrombus and cleaving the plasminogen via protease activity at residues 560–561 into the active form—plasmin—which then proteolytically destroys the fibrin clot [9]. This feature of tPA is used in thrombolytic therapy to treat ischemic stroke. In addition to the NINDS study, the clinical efficacy of tPA has been demonstrated in more recent studies [10,11,12].

tPA was first produced via recombinant biotechnological methods (PMID: 6337343). For pharmaceutical purposes, tPA was the first drug produced synthetically from mammalian cells, specifically Chinese hamster ovary cells. Recombinant tPA is sold under various brand names. There are some variants of tissue plasminogen activator, such as alteplase, reteplase [13], and tenecteplase. Reteplase and tenecteplase are the third generation of tPA drugs. However, reteplase is approved for thrombolytic treatment of acute myocardial infarction [14], whereas alteplase and tenecteplase are approved for acute ischemic stroke [6,15]. Alteplase has a very short circulating half-life and is, therefore, administered as a bolus (10% of the dose), followed by an infusion of the remaining drug over 1 h with rapid plasma concentrations that decrease in case of infusion delays of more than 5 min [16]. Tenecteplase is produced using recombinant DNA technology as a modification of alteplase. These modifications extend the half-life of Tenecteplase and increase affinity to fibrin and resistance to plasminogen activator inhibitor-1 [17]. Tenecteplase is administered as a single bolus, making it an attractive alternative to alteplase in the treatment of acute ischemic stroke [18].

Despite the efficacy of tPA being demonstrated in many papers, the controversy surrounding its use in ischemic stroke has not abated because of a number of disadvantages and side effects associated with this treatment. A significant disadvantage of thrombolytic therapy with tPA is the narrow therapeutic window of 4.5 h. The introduction of TPA outside of this time window increases the incidence of side effects [19]. In particular, the shorter the time between the onset of stroke symptoms and administration of tPA, the lower the incidences of repeated hospitalizations and mortality [20]. The therapeutic effect of TPA is inversely proportional to the time elapsed between the onset of stroke symptoms and the introduction of TPA [20,21]. Consequently, tPA is effective for no longer than 4.5 h after the onset of stroke symptoms, whereas the therapeutic window for mechanical thrombectomy is much wider, being 24 h after the onset of ischemia [22].

The pathological mechanisms induced by tPA may involve activation of neurotoxic concentrations of plasmin. The roles of plasminogen and plasmin have been described in the context of physiological neurobiology or pathological neurodegeneration. Plasmin has been found to have neurotoxic effects on neurons [23], and injection of plasmin into the striatum can lead to brain damage, increased infiltration of inflammatory cells into brain tissue, and a microglial response [24]. In contrast, plasminogen levels negatively correlated with brain injury when measured after thrombolysis with tPA after stroke [25]. Thus, the effects of plasmin and plasminogen on stroke and thrombolytic therapy appeared to be multidirectional, encompassing both negative effects of plasmin and putative positive effects of plasminogen, whereas the complexity of directly measuring endogenous plasmin dynamics in rodent brain tends to complicate the analysis of these mechanisms. In addition, there is evidence that tPA has a cytotoxic effect, enhances ischemic excitotoxicity, induces neuroinflammation and oxidative stress, increases the risk of orolingual angioedema, etc. [7,8,26,27]. However, one of the main side effects of tPA is hemorrhagic transformation, which occurs in 32% of cases, compared to only 20% without tPA [5]. The main reason for this effect is MMP activation in the ischemic tissue [28].

## 3. Hemorrhagic Transformation and Activation of MMP in Ischemic Stroke

Hemorrhagic transformation (HT) is a complication of ischemic stroke that results from a violation of the barrier function of blood vessels. According to the European Joint Acute Stroke Studies (ECASS) classification, HT is divided into hemorrhagic infarction—small hemorrhages at the edges of the focus—and parenchymal hematoma, which has a fused formation [5]. Both the development of the ischemic injury itself, which leads to a violation of the integrity of the BBB, and the introduction of thrombolytic agents, which leads to reperfusion, have a major impact [29]. Together with glutamate excitotoxicity, HT is a clinical manifestation of reperfusion injury [2]. The damage and increase in permeability of the BBB is related to the damage inflicted on the vascular endothelium and the destruction of its basement membrane [29,30], which is caused, among other factors, by the increased activity of MMPs, especially MMP-9 and MMP-2. Their proteolytic properties contribute to the destruction of the components of the BBB and increase its permeability. The effect of tPA on the activity of MMPs is apparently one of the main reasons for the increased risk of HT during thrombolytic therapy [31]. It has been shown that within 36 h of stroke onset, symptomatic intracerebral hemorrhage occurred in 6.4% of patients treated with tPA, whereas this outcome occurred in only 0.6% of patients treated with placebo [6].

There are also a number of studies that show activation of MMP when simulating ischemia–reperfusion injury in vitro under oxygen–glucose deprivation (OGD) [32,33,34]. In this model, cells are incubated in a solution without glucose under oxygen-free conditions and simultaneously reoxygenated with the return to a full culture medium. MMP-9 activity has been shown to increase in brain cell cultures during reoxygenation after OGD. Thus, astrocytes and endotheliocytes (bEnd line) showed a gradual increase in the MMP-9 activity in the cell supernatant after 90 min of OGD at 2, 4, 8, and 12 h. However, in primary neurons, MMP-9 activity was not detected and did not increase after OGD [35].

It was shown that the activity of MMP-2 and MMP-9 increased after 2 h of OGD in conditioned culture medium, while it decreased in cell lysates of bEnd endotheliocytes, C8-D1A astrocytes, and SH-SY5Y neuroblastoma cells. This finding indicates that OGD increases the secretion of these enzymes into the extracellular space [33]. It should be noted that MMPs are secreted into the extracellular space in the form of inactive pro-enzymes. The cleavage of pro-MMPs required for their conversion to the active form occurs in the extracellular space. Therefore, the zymograms of the conditioned culture medium and cell lysates may differ significantly with respect to the ratio of active and inactive forms of MMPs [36].

An increase in the activity of MMP-9 in the culture medium of the SH-SY5Y neuronal line is in contradiction with the data that suggest that the activity of MMP-9 is not detected in the supernatants of primary neuron cultures either in the normal state or after OGD. This phenomenon is probably related to the different origin of the cell cultures, since SH-SY5Y is a neuroblastoma cell line that exhibits a neuronal phenotype [37] but does not correspond to primary neurons. Moreover, the activity of various MMPs is a characteristic feature of many tumors, especially metastatic tumors.

Although there is evidence that OGD does not always increase the activity of MMP-2 and MMP-9 in the culture medium of bEnd cells [32], in endothelial cells, an increase in MMP activity after ischemia is virtually universally recognized.

While MMP-2 and MMP-9 are the most studied members of the matrix metalloproteinase family, there are data related to the increase in activity and expression of other MMPs, such as MMP-1, MMP-3, MMP-8, MMP-10, MMP-12, and MMP-13, during the pathology. In mice with bilateral common carotid arterial occlusion, MMP-1 increased in addition to MMP-2 [38], though its role remains unclear. MMP-13 has been shown to have a similar pattern to MMP-2 and MMP-9, i.e., it promotes HT in the acute phase of stroke and is involved in the regeneration of damaged tissue in the late phase [39]. The levels of MMP-8 and MMP-10 were increased after stroke and correlated with negative outcome [40,41,42]. Suppression of MMP-12 contributed to neurological recovery in rats after stroke [43], and its knockdown prevented secondary brain damage [44]. One study has shown that MMP-3 contributes to the induction of hemorrhagic transformations associated with t-PA treatment after ischemic stroke [45]. Moreover, in the above-mentioned study, a significant increase in MMP-3 expression that specifically occurred in ischemic hemisphere endothelial cells was detected after t-PA treatment. Interestingly, the increase in MMP-3 and MMP-13 expression after ischemia was observed in endothelial cells, MMP-12 was observed in activated microglia, and MMP-13 expression decreases in resting microglia in the infarcted area and undamaged neurons of the cerebral cortex [46].

## 4. The Dynamics of MMP-2 and MMP-9 Activity during Ischemic Stroke

Examination of the activity of MMP-2 and MMP-9 in protein extracts from human brain tissue at different time points after ischemic stroke revealed a transient dependence during the course of pathology. Brain tissue samples obtained at autopsy were examined from eight patients who died at different time points after stroke (from 2 days to 7 years). The activities of the different MMP types were compared in the stroke focus and a similar area of the healthy hemisphere. The results of zymography showed that MMP-9 activity was markedly increased in lesions in patients 2–4 days after stroke compared to intact brain areas, whereas there was virtually no change in MMP-2 activity [47]. In the patients whose autopsy material was analyzed several months or years after stroke, the activity of MMP-9 was increased to a much lesser extent in the foci, though, at the same time, increased activity of MMP-2 was observed [47]. These data suggest that the activity of MMP-2 and MMP-9 have different dynamics: MMP-9 is induced in the early post-stroke phase, whereas MMP-2 is induced much later.

**Figure 1:**
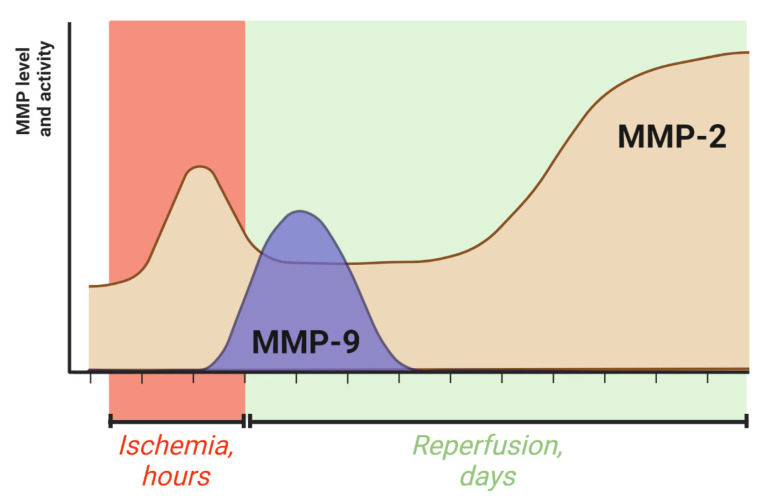
Dynamics of the level and activity of MMP-2 and MMP-9 in the brain during ischemic stroke.

Data obtained during the simulation of ischemic stroke in animals via middle cerebral artery occlusion (MCAO) showed that in the first hours after ischemic injury, the content and activity of MMP-9 increased significantly in the focal zone [48]. The dynamics of the ratio of MMP-9/pro-MMP-9 in the ischemic focus was also interesting. In the first few hours after ischemic injury, the level of pro-MMP-9 increased primarily, and one day later, the level of the active form increased. At day 4, the ratio of MMP-9/pro-MMP-9 decreased. In the period between 5 and 15 days, the MMP-9 concentration decreased to the basal level [48]. At the same time, an increase in the level of MMP-9 in the peri-infarct zone was not observed until 7–14 days after ischemia [49]. It has been shown that foci of functional recovery of brain tissue occurred in this zone, in which MMP-9 was required for extracellular matrix remodeling [49]. Thus, MMP-9 was initially activated in the ischemic area, and as the brain recovered, MMP-9 activity decreased in the ischemic foci and shifted to the peri-infarct area. As a result of analysis of a number of experimental studies and clinical data, we concluded that the activities of the two important MMPs have complex dynamics. In the acute phase of cerebral ischemia, according to experimental animal models, the dynamics of MMP-2 activity differ from that of MMP-9 activity. These data, in combination with the results of the clinical studies and autopsy findings described above, highlight the biphasic nature of the changes in the two MMPs (Figure 1).

The initial increase in MMP-2 activity in the brain apparently begins during ischemia because there is an increase in activity in the interstitial space of the brain immediately after the end of MCAO and before the onset of reperfusion. The increase in activity of MMP-9 in the interstitial space of the brain immediately after ischemia is not as pronounced. Analysis of MMP-2/9 activity in the interstitial space was performed via zymography in samples obtained during microdialysis of the brain in vivo [33]. Interestingly, when ischemia is simulated in the bEnd endothelial line, an increase in MMP-2 and MMP-9 activity is observed at the end of 2-h OGD and at the beginning of reoxygenation [33]. One of the possible explanations for this phenomenon is that the increase in MMP-2 activity in the extracellular space of the brain during this period is due to the production of this enzyme by multiple cell types, rather than only by endothelial cells, and the dynamics of production in other cell types are different from the dynamics of endothelial cells. It has been shown that after 50 min of ischemia and 4 h of reperfusion, increased activity of MMP-2 can be observed [48]. However, the increased MMP-2 activity in rat brain was not observed after 24 h of reperfusion [48]. Thus, the initial short-term activation of MMP-2 is observed immediately after ischemic injury and subsides within 1 day [48]. In the long term, MMP-2 activity increases after stroke and remains elevated over a long period of time (months and years), as proven by the results in humans [47]. This finding is consistent with the fact that a very significant increase in MMP-2 was observed in rats four days after experimental stroke [48]. However, there are currently no clear data on the dynamics of MMP-2 activity and secretion in relation to the ischemic focus during ischemic stroke.

The dynamics of MMP-2 and MMP-9 activity reflect their multifunctional roles in stroke. Their influence has been shown to depend largely on the stage of pathology development, and activation of the same MMPs at different stroke stages can have both negative and regenerative effects [31].

## 5. Mechanisms of the Influence of tPA on MMPs

**Figure 2:**
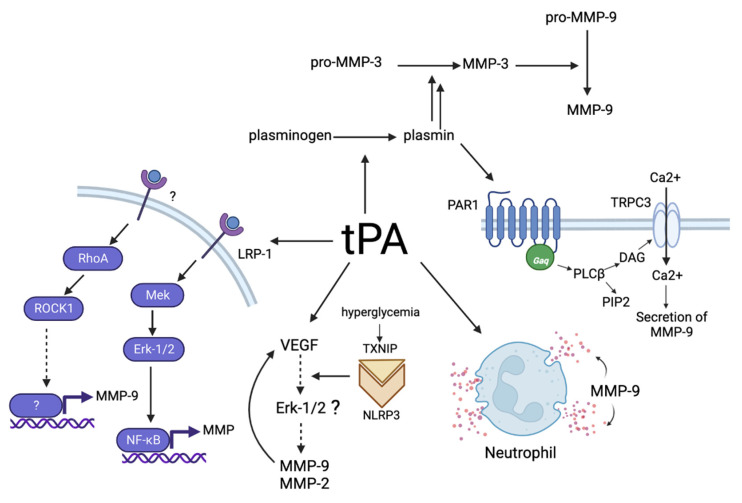
Mechanisms related to the influence of tPA on MMPs. tPA interacts with low-density lipoprotein receptor-related proteins (LRP) to activate intracellular signaling pathways, ultimately leading to increased MMP activity. Moreover, tPA activates the plasminogen-mediated proteolytic cascade, culminating in MMP activation. In addition, tPA has been shown to interact directly with MMPs, leading to their activation. It has been demonstrated that tPA can induce degranulation of neutrophils, whose granules are known to contain MMPs. Under conditions of hyperglycemia, tPA triggers TXNIP–NLRP3 inflammatory signaling. TXNIP binds to the NLRP3 protein and activates a post-ischemic inflammatory process that affects VEGF signaling, which, in turn, increases MMP levels (question mark indicates putative mechanism). Formation of plasmin based on the activity of tPA triggers activation of the PAR1 receptor, which relays a signal via the G protein Gαq that leads to the release of pro-MMP-9.

A tremendous accumulation of data contributed to our current understanding of the effects of tPA on the ischemic brain. As mentioned previously, the deleterious effects of tPA can largely be attributed to its ability to activate MMPs, whose expression and activity increase during ischemic injury. This result has been demonstrated both in animal models of brain ischemia and in cell cultures exposed to OGD [32,35,50,51,52,53]. The established mechanisms of the influence of tPA on MMPs are shown in Figure 2.

A. The involvement of tPA in the cascade of MMP activation due to its protease activity.

The ability of tPA to activate MMPs is closely related to its proteolytic activity. Through its primary physiological function of cleaving plasminogen to plasmin, tPA can initiate the activation of pro-MMP-3, which is the precursor of MMP-3, to its active form through proteolysis. Once MMP-3 is activated, it can cleave pro-MMP-9 to MMP-9 [28]. Consequently, tPA has the potential to significantly increase the activity of both MMP-3 and MMP-9 through direct proteolysis. This mechanism is of critical importance in the development of ischemic injury. MMP-3 can lead to the degradation of basal lamina and extracellular matrix proteins, while MMP-9 is involved in promoting edema and disrupting the BBB [28,31]. Therefore, the ability of tPA to activate MMP-3 and MMP-9 may exacerbate the progression of ischemic injury.

B. Increased MMPs gene expression.

Through its interactions with various receptors, tPA triggers a cascade of intracellular signaling pathways that promote MMP gene expression [54,55]. It is known that tPA increases the expression of MMP-2 and MMP-9 genes in both glial and endothelial cells, as shown by previous studies [56,57,58].

C. tPA-induced neutrophil degranulation with the release of MMP-9.

Neutrophils play an essential role in the inflammatory response and may release their secretory granules, which contain MMP-9, which is necessary for the degradation of the extracellular matrix and basal plate during infiltration into tissues [59]. It has been shown that after incubation of neutrophils with tPA, the level of MMP-9 increases rapidly in the culture medium and decreases in the cell lysates, which is due to the release from the secretory granules [29,60,61]. Remarkably, MMP-9 is found in neutrophil granules, whereas MMP-2 is not expressed in these cells. This result is further evidence of the separate and distinct functions of the different MMPs in the context of ischemic stroke pathophysiology [62].

D. Effect of tPA on MMP-9 secretion.

The effects of tPA on short-term secretion of MMP-9 have been extensively studied in endothelial cells. The mechanism involves the formation of plasmin via the activity of tPA, which, in turn, triggers activation of the PAR1 receptor. Activation of PAR1 then transmits a signal through the G protein Gαq, which activates TRPC3 channels through PLCβ and diacylglycerol, allowing Ca^2+^ ions to be transported into the cell. This process leads to an increased intracellular Ca^2+^ concentration, which eventually results in the secretion of pro-MMP-9. Notably, the concomitant influx of Na^+^ depolarizes the plasma membrane, which can suppress Ca^2+^ influx, leading to negative regulation of Ca-dependent MMP-9 secretion. This dynamic interplay between Ca^2+^ and Na^+^ fluxes results in short-term pulsed MMP-9 secretion in response to tPA stimulation [63]. Interestingly, inhibition of this regulatory mechanism by glibenclamide has been shown to have beneficial effects in patients with ischemic stroke [63].

E. Increased VEGF levels.

It has been shown that tPA increases VEGF levels in rats in the area of ischemic brain injury after MCAO for 4.5 h [64]. There is evidence that the tPA-mediated increase in VEGF levels during ischemia can be prevented by inhibitors of the Akt and RhoA signaling molecules [65]. VEGF increases the levels of MMP-2 and MMP-9, although the molecular mechanism of this phenomenon is not well understood. It is known that VEGF can activate the Erk-1/2 pathway [66]. On the other hand, the Erk-1/2 pathway regulates the expression of MMP-9 and MMP-2 [67,68]. Therefore, VEGF is expected to increase the expression of these MMPs via the MAPK/Erk-1/2 pathway. Interestingly, tPA triggers the TXNIP-NLRP3 inflammatory signaling pathway under conditions of hyperglycemia, which is a common accompaniment of ischemic stroke. The TXNIP protein, which is sensitive to intracellular glucose levels, is activated during hyperglycemia and under conditions of oxidative stress that are exacerbated by tPA. TXNIP binds to NLRP3 protein and activates a post-ischemic inflammatory process that affects VEGF signaling, which, in turn, increases MMP levels. Thus, hyperglycemia enhances the tPA-induced increase in MMP levels [69].

F. Receptors and signaling pathways mediating tPA-induced MMP expression.

tPA does not have its own specific cell surface receptors. However, it can interact with a number of non-specific receptors. The best known examples of these receptors are LRP-1 and annexin A2 [70]. There is also evidence that tPA can bind to glutamate NMDA receptors, EGF, and mannose receptors. Interaction with these receptors mediates various physiological effects of tPA [71,72,73], though LRP-1 apparently plays the major role in tPA-induced activation of MMPs [74].

The interaction of tPA with LRP-1 leads to the activation of the transcription factor NF-kB [27], which, among other effects, induces MMP gene expression, especially MMP-3 [55]. It has been shown that binding of tPA to LRP-1 also leads to a decrease in transcription of TAP mRNA, i.e., the formation of a negative feedback loop that enables the control of tPA biosynthesis [75].

Apparently, Mek1/Erk-1/2 kinases, which function as intermediate signaling kinases of a cascade [54,76] in a similar way to the HGF signal transduction mechanism with kringle domains, such as tPA, play an important role in signal transduction from LRP-1 to NF-kB. This observation is an important argument in favor of the function of tPA as a cytokine [54].

Of great interest is a study that indicates that ischemia increases the expression of LRP in endothelial cells, which enhances signaling upon exposure to tPA [32]. This potential outcome would generally suggest that the effect of tPA is enhanced under conditions of ischemia.

G. The contribution of Rho-kinases.

A number of studies point to a possible link between the hemorrhagic transformation induced via exposure to tPA during ischemic stroke and an increase in the expression of Rho kinases [62,77]. These kinases regulate contractility, mobility, and proliferation of many cell types tat are important in the pathogenesis of ischemic injury and recovery after stroke. In the 1-hour rat OSMA model, the expression of Rho kinases in the ischemic area gradually increased during 48 h of reperfusion [78]. At the same time, inhibition of Rho kinases positively affected the outcome of stroke in the MCAO model by reducing the size of the damaged area and improving neurological functions. In this regard, Rho kinases can be considered as potential targets for the treatment of ischemic stroke [79].

Recently, tPA was shown to increase the activity of pMLC and cofilin, which are direct targets of Rho kinase phosphorylation [62]. It was also found that the Rho kinase inhibitor Y27632 suppresses the expression of the MMP-9 gene in vascular smooth muscle cells. However, this effect is not associated with the major signaling pathways that control MMP-9 expression, as Y27632 did not affect the activity of the major participants in these pathways, particularly Erk and NF-kB. Therefore, the authors suggest that the activities of Rho kinases are associated with a different way of regulating MMP-9 gene expression [80].

## 6. Ways to Reduce MMP Activation in Ischemic Stroke

Currently, new therapeutic approaches intended to reduce the adverse effects of ischemic stroke are being actively sought and researched, including inhibitors of MMP activity. It is worth noting that the therapeutic efficacy of MMP inhibition depends on the time elapsed after ischemia. This observation is true due to the differences in MMP functions at the different stages of stroke development described earlier in this paper. Thus, inhibition of MMP-9 on the first day after stroke has a positive effect on its outcome; on the third day, there is no neuroprotective effect; and on the seventh day, it leads to an increase in damage [49].

There are a number of approaches that aim to alter MMP expression and activity that have shown high efficacy in pre-clinical studies [81]. Among the most promising areas in this field are the following topics.

Minocycline is a second-generation antibiotic from the tetracycline group that inhibits MMP activity, as well as having antibacterial activity [82,83]. Minocycline is also known to be an effective adjuvant in thrombolytic therapy of ischemic stroke. In rats subjected to MCAO, combination therapy using tPA and minocycline for 6 h was shown to reduce the amount of MMP-9 in plasma, decrease the volume of ischemic damage, and decrease intracranial hemorrhage compared to administration of tPA alone. Such combination therapy has also been shown to attenuate neutrophil infiltration and microglial activation, reduce the amount of active MMP-9, and decrease the degradation of the tight junction protein claudin-5 in the peri-infarct zone [84]. In the first and second phases of clinical trials, minocycline alone was shown to be safe in the treatment of ischemic stroke [85]. However, it is worth awaiting the results of several other larger clinical trials with minocycline that are currently underway.

Otaplimastat, which is also known as SP-8203, is a small molecule with a quinazoline-2,4-dione scaffold. It improves neurological outcomes in various animal stroke models through multiple cytoprotective mechanisms. In embolic stroke models, otaplimastat has shown significant benefit in terms of reducing infarct volume and edema, both as a single treatment and in combination with tPA [86,87]. In the rat embolic middle cerebral artery occlusion model, otaplimastat reduced cerebral infarct size and edema and improved neurological function. It also prolonged the therapeutic time window of tPA by reducing the intracerebral hemorrhagic transformation and mortality triggered due to delayed tPA treatment. Ischemia-induced MMP expression was closely correlated with cerebral hemorrhagic transformation and brain injury, making otaplimastat a potentially potent inhibitor of stroke injury when administered in combination with tPA. In the OGD model of endothelial cells, otaplimastat suppresses the activity of MMPs by restoring TIMP levels and reducing vascular permeation [88]. In a phase-1 clinical trial, otaplimastat at doses of up to 240 mg was well tolerated by 77 healthy volunteers, with no significant side effects recorded. In a two-part and multicenter phase-2 study of stroke patients receiving tPA, otaplimastat was administered intravenously <30 min after tPA. The study found that otaplimastat adjunctive therapy in patients treated with tPA was feasible and generally safe, with no safety issues recorded in either phase. However, the functional efficacy of otaplimastat needs to be investigated in further large studies [89].

Epigallocatechin gallate (EGCG), which is also known as epigallocatechin-3-gallate, is a type of catechin. Indeed, it is the most abundant catechin in green tea. As a polyphenol, EGCG is currently being investigated in basic research for its potential to influence human health and disease [90,91]. The neuroprotective efficacy of EGCG has been investigated by modeling focal cerebral ischemia in rats [92]. Forelimb function was significantly improved in the EGCG-treated group compared to the MCAO control group, with normal function achieved by day 10 and improvements sustained until day 14 post-ischemia. However, infarct volume did not differ between groups, and hindlimb function was not affected [93]. The effect of EGCG on prolonging the therapeutic window of tPA was studied in an MCAO model in rats. The common side effects of delayed tPA treatment, including cerebral infarction, cerebral edema, and blood–brain barrier disruption, were significantly reduced by EGCG. In the brain, EGCG was shown to upregulate plasminogen activator inhibitor (PAI-1) expression and downregulate MMP-2 and MMP-9 expression [94]. The purpose of a randomized, double-blind, and placebo-controlled trial was to investigate whether the additional administration of EGCG to treat stroke patients with tPA could extend their narrow therapeutic window. The study enrolled 429 patients with acute ischemic stroke who met inclusion criteria, i.e., a well-defined time of onset, a measurable deficit on the National Institutes of Health Stroke Scale (NIHSS), and a CT scan without intracranial hemorrhage. Patients were randomly assigned to treatment with either tPA and EGCG or placebo, and outcomes were measured via NIHSS and plasma levels of MMP-2 and 9. EGCG administration significantly improved treatment outcomes in delayed-onset patients, possibly by lowering plasma MMP-2 and 9 levels, as shown based on strong linear correlations with NIHSS scores. The authors concluded that EGCG could potentially complement conventional tPA treatment to extend the narrow therapeutic window and improve outcomes related to the treatment of late stroke [95].

Stem cells. Recently, a growing body of data has been collected regarding the efficacy of cell therapy based on mesenchymal stromal cells (MSCs) in various diseases, including ischemic stroke. In particular, MSCs have been shown to improve BBB integrity in this pathology [70,96,97]. A number of studies have shown that the activity of MMP-9 decreases when MSCs are transplanted into the MCAO model and MSCs are co-cultured with endothelial cells subjected to OGD [98], whereas the activity of MMP-2 does not change. Presumably, this therapeutic effect of MSCs is due to the suppression of the expression of intercellular adhesion molecules (ICAM) involved in the migration of leukocytes, including neutrophils [99]. As mentioned previously, neutrophils are the source of MMP-9, and exposure to tPA can trigger degranulation of these cells. Thus, the ability of MSCs to improve the integrity of the BBB in ischemic stroke could be explained in part by a reduction in the infiltration of neutrophils into the lesion and, consequently, the reduced release of MMP-9 from these cells. In this regard, cell therapy might have a protective effect when administered together with tPA in ischemic stroke. However, there is evidence that MSCs themselves may increase the expression of tPA in neurons and astrocytes via the endogenous Hedgehog signaling pathway [100,101]. In addition, MSCs have been shown to secrete tissue inhibitors of matrix metalloproteinases 1 and 2 (TIMP 1–2), which exert an inhibitory effect on MMPs. This inhibition may prevent degradation of the extracellular matrix after ischemic stroke, thereby preventing further damage to the BBB [102,103]. It is also possible to use MSCs that overexpress TIMP-1 [104] for more effective treatment of tPA-associated brain injury.

Lithium salts. Lithium salts are conventionally used to treat bipolar disorder [105]. A number of studies have shown their efficacy in reducing the volume of ischemic damage and cerebral edema and improving the integrity of the BBB [106,107,108]. It has also been shown that pre-conditioning astrocyte and endothelial cell cultures with lithium chloride increases their survival in OGD [109]. Moreover, these effects have been associated with a decrease in MMP-9 activity in the ischemic brain [106,107]. Currently, it is not entirely clear how LiCl reduces MMP-9 activity. It has been suggested that the lithium-induced decrease in MMP-9 activity is related to the activation of the MAPK/Erk-1/2 pathway, as lithium increases the phosphorylation of its participants [106]. We have already mentioned that this pathway is a positive regulator of MMP-9 gene expression; thus, this hypothesis leads to a contradiction and requires additional analyzes. Moreover, the beneficial effect of lithium on BBB integrity after ischemia may be due to its ability to regulate the Wnt/β-catenin signaling pathway by inhibiting GSK-3ß kinase. This signaling pathway is known to be important for BBB formation and the synthesis of dense contact proteins [107]. Therefore, it can be assumed that lithium ions may have a protective effect in the treatment of tPA brain ischemia. 

## 7. Conclusions

The outcome of thrombolytic therapy based on the administration of tPA depends largely on the modulation of the activity of different types of MMPs. tPA increases the level and activity of MMPs in brain tissue via different mechanisms and enhances their adverse effects during the acute phase of stroke. Different types of MMPs have different effects on the ischemic brain, and their concentration, activity, and localization vary depending on the time elapsed since injury. When tPA is administered outside of the therapeutic window, the increase in certain MMPs that it causes coincides with the peak of their post-ischemic activity in the brain, resulting in damage to the blood–brain barrier and a marked increase in the risk of hemorrhagic transformation. This outcome is most pronounced for MMP-9, whose activity dynamics in the region of injury seems to determine the limit of the therapeutic window for tPA administration. Understanding these mechanisms underscores the need to search for additional therapeutic agents to extend the therapeutic window and reduce the risk of hemorrhagic transformation during thrombolytic therapy. An effective solution to this problem could be the use of specific MMP inhibitors. However, because of the multifunctionality and complex dynamics of MMP activation, this method might only be effective in a certain period of time; therefore, it requires careful study of the action plan to maintain adequate MMP function during the different phases of stroke rehabilitation.

## Data Availability

Data is available from the corresponding authors upon reasonable request.

## References

[1] Sacco R.L., Kasner S.E., Broderick J.P., Caplan L.R., Connors J.J., Culebras A., Elkind M.S.V., George M.G., Hamdan A.D., Higashida R.T. (2013). An Updated Definition of Stroke for the 21st Century: A Statement for Healthcare Professionals from the American Heart Association/American Stroke Association. Stroke.

[2] Campbell B.C.V., Khatri P. (2020). Stroke. Lancet.

[3] Dewar B., Shamy M. (2020). TPA for Acute Ischemic Stroke and Its Controversies: A Review. Neurohospitalist.

[4] Powers W.J., Rabinstein A.A., Ackerson T., Adeoye O.M., Bambakidis N.C., Becker K., Biller J., Brown M., Demaerschalk B.M., Hoh B. (2018). 2018 Guidelines for the Early Management of Patients with Acute Ischemic Stroke: A Guideline for Healthcare Professionals from the American Heart Association/American Stroke Association. Stroke.

[5] Honig A., Percy J., Sepehry A.A., Gomez A.G., Field T.S., Benavente O.R. (2022). Hemorrhagic Transformation in Acute Ischemic Stroke: A Quantitative Systematic Review. J. Clin. Med..

[6] NINDS rt-PA Stroke Study Group (1995). NINDS RTPA: Tissue Plasminogen Activator for Acute Ischemic Stroke. N. Engl. J. Med..

[7] Chapman S.N., Mehndiratta P., Johansen M.C., McMurry T.L., Johnston K.C., Southerland A.M. (2014). Current Perspectives on the Use of Intravenous Recombinant Tissue Plasminogen Activator (TPA) for Treatment of Acute Ischemic Stroke. Vasc. Health Risk Manag..

[8] Hébert M., Lesept F., Vivien D., Macrez R. (2016). The Story of an Exceptional Serine Protease, Tissue-Type Plasminogen Activator (TPA). Rev. Neurol..

[9] Collen D., Lijnen H.R. (2004). Tissue-Type Plasminogen Activator: A Historical Perspective and Personal Account. J. Thromb. Haemost..

[10] Kwiatkowski T.G., Libman R.B., Frankel M., Tilley B.C., Morgenstern L.B., Lu M., Broderick J.P., Lewandowski C.A., Marler J.R., Levine S.R. (1999). Effects of Tissue Plasminogen Activator for Acute Ischemic Stroke at One Year. National Institute of Neurological Disorders and Stroke Recombinant Tissue Plasminogen Activator Stroke Study Group. N. Engl. J. Med..

[11] Schwammenthal Y., Drescher M.J., Merzeliak O., Tsabari R., Bruk B., Feibel M., Hoffman C., Bakon M., Rotstein Z., Chapman J. (2004). Intravenous Recombinant Tissue Plasminogen Activator Therapy for Acute Ischemic Stroke: Initial Israeli Experience. Age.

[12] Wardlaw J.M., Murray V., Berge E., del Zoppo G.J. (2014). Thrombolysis for Acute Ischaemic Stroke. Cochrane Database Syst. Rev..

[13] Pennica D., Holmes W.E., Kohr W.J., Harkins R.N., Vehar G.A., Ward C.A., Bennett W.F., Yelverton E., Seeburg P.H., Heyneker H.L. (1983). Cloning and Expression of Human Tissue-Type Plasminogen Activator CDNA in *E. coli*. Nature.

[14] Global Use of Strategies to Open Occluded Coronary Arteries (GUSTO III) Investigators (1997). A Comparison of Reteplase with Alteplase for Acute Myocardial Infarction. N. Engl. J. Med..

[15] Van de Werf F., Adgey J., Ardissino D., Armstrong P.W., Aylward P., Barbash G., Betriu A., Binbrek A.S., Califf R., Diaz R. (1999). Single-Bolus Tenecteplase Compared with Front-Loaded Alteplase in Acute Myocardial Infarction: The ASSENT-2 Double-Blind Randomised Trial. Lancet.

[16] Smith C., Al-Nuaimi Y., Wainwright J., Sherrington C., Singh A., Kallingal J., Douglass C., Parry-Jones A., Smith C., Dixit A. (2012). The Influence of Bolus to Infusion Delays on Plasma Tissue Plasminogen Activator Levels. Int. J. Stroke.

[17] Nordt T.K., Bode C. (2003). Thrombolysis: Newer Thrombolytic Agents and Their Role in Clinical Medicine. Heart.

[18] Potla N., Ganti L. (2022). Tenecteplase vs. Alteplase for Acute Ischemic Stroke: A Systematic Review. Int. J. Emerg. Med..

[19] Broderick J.P. (1997). Intracerebral Hemorrhage After Intravenous T-PA Therapy for Ischemic Stroke. Stroke.

[20] Man S., Xian Y., Holmes D.N., Matsouaka R.A., Saver J.L., Smith E.E., Bhatt D.L., Schwamm L.H., Fonarow G.C. (2020). Association between Thrombolytic Door-to-Needle Time and 1-Year Mortality and Readmission in Patients with Acute Ischemic Stroke. JAMA J. Am. Med. Assoc..

[21] Knecht T., Borlongan C., Peña I. (2018). Combination Therapy for Ischemic Stroke: Novel Approaches to Lengthen Therapeutic Window of Tissue Plasminogen Activator. Brain Circ..

[22] Yang S.H., Liu R. (2021). Four Decades of Ischemic Penumbra and Its Implication for Ischemic Stroke. Transl. Stroke Res..

[23] Wu J., Echeverry R., Guzman J., Yepes M. (2010). Neuroserpin Protects Neurons from Ischemia-Induced Plasmin-Mediated Cell Death Independently of Tissue-Type Plasminogen Activator Inhibition. Am. J. Pathol..

[24] Xue M., Del Bigio M.R. (2001). Acute Tissue Damage after Injections of Thrombin and Plasmin into Rat Striatum. Stroke.

[25] Sun X., Berthiller J., Derex L., Trouillas P., Diallo L., Hanss M. (2015). Post-Thrombolysis Haemostasis Changes after Rt-PA Treatment in Acute Cerebral Infarct. Correlations with Cardioembolic Aetiology and Outcome. J. Neurol. Sci..

[26] Wang F.J., Wang S.X., Chai L.J., Zhang Y., Guo H., Hu L.M. (2018). Xueshuantong Injection (Lyophilized) Combined with Salvianolate Lyophilized Injection Protects against Focal Cerebral Ischemia/Reperfusion Injury in Rats through Attenuation of Oxidative Stress. Acta Pharmacol. Sin..

[27] Wei C.C., Kong Y.Y., Hua X., Li G.Q., Zheng S.L., Cheng M.H., Wang P., Miao C.Y. (2017). NAD Replenishment with Nicotinamide Mononucleotide Protects Blood-Brain Barrier Integrity and Attenuates Delayed Tissue Plasminogen Activator-Induced Haemorrhagic Transformation after Cerebral Ischaemia. Br. J. Pharmacol..

[28] Kurzepa J., Kurzepa J., Golab P., Czerska S., Bielewicz J. (2014). The Significance of Matrix Metalloproteinase (MMP)-2 and MMP-9 in the Ischemic Stroke. Int. J. Neurosci..

[29] Leira R., Sobrino T., Blanco M., Campos F., Rodríguez-Ýñez M., Castellanos M., Moldes O., Millán M., Dávalos A., Castillo J. (2012). A Higher Body Temperature Is Associated with Haemorrhagic Transformation in Patients with Acute Stroke Untreated with Recombinant Tissue-Type Plasminogen Activator (RtPA). Clin. Sci..

[30] Álvarez-Sabín J., Maisterra O., Santamarina E., Kase C.S. (2013). Factors Influencing Haemorrhagic Transformation in Ischaemic Stroke. Lancet Neurol..

[31] Montaner J., Ramiro L., Simats A., Hernández-Guillamon M., Delgado P., Bustamante A., Rosell A. (2019). Matrix Metalloproteinases and ADAMs in Stroke. Cell. Mol. Life Sci..

[32] Cheon S.Y., Kim S.Y., Kam E.H., Lee J.H., Kim J.M., Kim E.J., Kim T.W., Koo B.N. (2017). Isoflurane Preconditioning Inhibits the Effects of Tissue-Type Plasminogen Activator on Brain Endothelial Cell in an in Vitro Model of Ischemic Stroke. Int. J. Med. Sci..

[33] Liu J., Jin X., Liu K.J., Liu W. (2012). Matrix Metalloproteinase-2-Mediated Occludin Degradation and Caveolin-1-Mediated Claudin-5 Redistribution Contribute to Blood-Brain Barrier Damage in Early Ischemic Stroke Stage. J. Neurosci..

[34] Neuhaus W., Gaiser F., Mahringer A., Franz J., Riethmüller C., Förster C. (2014). The Pivotal Role of Astrocytes in an in Vitro Stroke Model of the Blood-Brain Barrier. Front. Cell. Neurosci..

[35] Kenna J.E., Anderton R.S., Knuckey N.W., Meloni B.P. (2020). Assessment of Recombinant Tissue Plasminogen Activator (RtPA) Toxicity in Cultured Neural Cells and Subsequent Treatment with Poly-Arginine Peptide R18D. Neurochem. Res..

[36] Murphy G., Stanton H., Cowell S., Butler G., Knäuper V., Atkinson S., Gavrilovic J. (1999). Mechanisms for pro Matrix Metalloproteinase Activation. APMIS.

[37] Kovalevich J., Langford D. (2013). Considerations for the Use of SH-SY5Y Neuroblastoma Cells in Neurobiology. Methods Mol. Biol..

[38] Victoria E.C.G., de Brito Toscano E.C., Oliveira F.M.S., de Carvalho B.A., Caliari M.V., Teixeira A.L., de Miranda A.S., Rachid M.A. (2020). Up-Regulation of Brain Cytokines and Metalloproteinases 1 and 2 Contributes to Neurological Deficit and Brain Damage in Transient Ischemic Stroke. Microvasc. Res..

[39] Ma F., Martínez-San Segundo P., Barceló V., Morancho A., Gabriel-Salazar M., Giralt D., Montaner J., Rosell A. (2016). Matrix Metalloproteinase-13 Participates in Neuroprotection and Neurorepair after Cerebral Ischemia in Mice. Neurobiol. Dis..

[40] Rodríguez J.A., Sobrino T., Orbe J., Purroy A., Martínez-Vila E., Castillo J., Páramo J.A. (2013). ProMetalloproteinase-10 Is Associated with Brain Damage and Clinical Outcome in Acute Ischemic Stroke. J. Thromb. Haemost..

[41] Cuadrado E., Rosell A., Penalba A., Slevin M., Alvarez-Sabín J., Ortega-Aznar A., Montaner J. (2009). Vascular MMP-9/TIMP-2 and Neuronal MMP-10 up-Regulation in Human Brain after Stroke: A Combined Laser Microdissection and Protein Array Study. J. Proteome Res..

[42] Palm F., Pussinen P.J., Safer A., Tervahartiala T., Sorsa T., Urbanek C., Becher H., Grau A.J. (2018). Serum Matrix Metalloproteinase-8, Tissue Inhibitor of Metalloproteinase and Myeloperoxidase in Ischemic Stroke. Atherosclerosis.

[43] Challa S.R., Nalamolu K.R., Fornal C.A., Wang B.C., Martin R.C., Olson E.A., Ujjainwala A.L., Pinson D.M., Klopfenstein J.D., Veeravalli K.K. (2022). Therapeutic Efficacy of Matrix Metalloproteinase-12 Suppression on Neurological Recovery after Ischemic Stroke: Optimal Treatment Timing and Duration. Front. Neurosci..

[44] Arruri V., Chokkalla A.K., Jeong S., Chelluboina B., Mehta S.L., Veeravalli K.K., Vemuganti R. (2022). MMP-12 Knockdown Prevents Secondary Brain Damage after Ischemic Stroke in Mice. Neurochem. Int..

[45] Suzuki Y., Nagai N., Umemura K., Collen D., Lijnen H.R. (2007). Stromelysin-1 (MMP-3) Is Critical for Intracranial Bleeding after t-PA Treatment of Stroke in Mice. J. Thromb. Haemost..

[46] Hohjoh H., Horikawa I., Nakagawa K., Segi-Nishida E., Hasegawa H. (2020). Induced MRNA Expression of Matrix Metalloproteinases Mmp-3, Mmp-12, and Mmp-13 in the Infarct Cerebral Cortex of Photothrombosis Model Mice. Neurosci. Lett..

[47] Clark A.W., Krekoski C.A., Bou S.S., Chapman K.R., Edwards D.R. (1997). Increased Gelatinase A (MMP-2) and Gelatinase B (MMP-9) Activities in Human Brain after Focal Ischemia. Neurosci. Lett..

[48] Planas A.M., Solé S., Justicia C. (2001). Expression and Activation of Matrix Metalloproteinase-2 and -9 in Rat Brain after Transient Focal Cerebral Ischemia. Neurobiol. Dis..

[49] Zhao B.Q., Wang S., Kim H.Y., Storrie H., Rosen B.R., Mooney D.J., Wang X., Lo E.H. (2006). Role of Matrix Metalloproteinases in Delayed Cortical Responses after Stroke. Nat. Med..

[50] Lapchak P.A., Chapman D.F., Zivin J.A. (2000). Metalloproteinase Inhibition Reduces Thrombolytic (Tissue Plasminogen Activator)-Induced Hemorrhage after Thromboembolic Stroke. Stroke.

[51] Pfefferkorn T., Rosenberg G.A. (2003). Closure of the Blood-Brain Barrier by Matrix Metalloproteinase Inhibition Reduces RtPA-Mediated Mortality in Cerebral Ischemia with Delayed Reperfusion. Stroke.

[52] Sumii T., Lo E.H. (2002). Involvement of Matrix Metalloproteinase in Thrombolysis-Associated Hemorrhagic Transformation after Embolic Focal Ischemia in Rats. Stroke.

[53] Wang X., Lee S.R., Arai K., Lee S.R., Tsuji K., Rebeck G.W., Lo E.H. (2003). Lipoprotein Receptor-Mediated Induction of Matrix Metalloproteinase by Tissue Plasminogen Activator. Nat. Med..

[54] Hu K., Yang J., Tanaka S., Gonias S.L., Mars W.M., Liu Y. (2006). Tissue-Type Plasminogen Activator Acts as a Cytokine That Triggers Intracellular Signal Transduction and Induces Matrix Metalloproteinase-9 Gene Expression. J. Biol. Chem..

[55] Suzuki Y., Nagai N., Yamakawa K., Kawakami J., Lijnen H.R., Umemura K. (2009). Tissue-Type Plasminogen Activator (t-PA) Induces Stromelysin-1 (MMP-3) in Endothelial Cells through Activation of Lipoprotein Receptor-Related Protein. Blood.

[56] Song H., Cheng Y., Bi G., Zhu Y., Jun W., Ma W., Wu H. (2016). Release of Matrix Metalloproteinases-2 and 9 by S-Nitrosylated Caveolin-1 Contributes to Degradation of Extracellular Matrix in TPA-Treated Hypoxic Endothelial Cells. PLoS ONE.

[57] Zhang C., An J., Haile W.B., Echeverry R., Strickland D.K., Yepes M. (2009). Microglial Low-Density Lipoprotein Receptor-Related Protein 1 Mediates the Effect of Tissue-Type Plasminogen Activator on Matrix Metalloproteinase-9 Activity in the Ischemic Brain. J. Cereb. Blood Flow Metab..

[58] Siniatchkin M., Averkina N., Andrasik F., Stephani U., Gerber W.D. (2006). Neurophysiological Reactivity before a Migraine Attack. Neurosci. Lett..

[59] Turner R.J., Sharp F.R. (2016). Implications of MMP9 for Blood Brain Barrier Disruption and Hemorrhagic Transformation Following Ischemic Stroke. Front. Cell. Neurosci..

[60] Cuadrado E., Ortega L., Hernández-Guillamon M., Penalba A., Fernández-Cadenas I., Rosell A., Montaner J. (2008). Tissue Plasminogen Activator (t-PA) Promotes Neutrophil Degranulation and MMP-9 Release. J. Leukoc. Biol..

[61] Gautier S., Ouk T., Tagzirt M., Lefebvre C., Laprais M., Pétrault O., Dupont A., Leys D., Bordet R. (2014). Impact of the Neutrophil Response to Granulocyte Colony-Stimulating Factor on the Risk of Hemorrhage When Used in Combination with Tissue Plasminogen Activator during the Acute Phase of Experimental Stroke. J. Neuroinflamm..

[62] Pan X.W., Wang M.J., Gong S.S., Sun M.H., Wang Y., Zhang Y.Y., Li F., Yu B.Y., Kou J.P. (2020). YiQiFuMai Lyophilized Injection Ameliorates TPA-Induced Hemorrhagic Transformation by Inhibiting Cytoskeletal Rearrangement Associated with ROCK1 and NF-ΚB Signaling Pathways. J. Ethnopharmacol..

[63] Gerzanich V., Kwon M.S., Woo S.K., Ivanov A., Marc Simard J. (2018). SUR1-TRPM4 Channel Activation and Phasic Secretion of MMP-9 Induced by TPA in Brain Endothelial Cells. PLoS ONE.

[64] Won S., Lee J.H., Wali B., Stein D.G., Sayeed I. (2014). Progesterone Attenuates Hemorrhagic Transformation after Delayed TPA Treatment in an Experimental Model of Stroke in Rats: Involvement of the VEGF–MMP Pathway. J. Cereb. Blood Flow Metab..

[65] Wang L., Fan W., Cai P., Fan M., Zhu X., Dai Y., Sun C., Cheng Y., Zheng P., Zhao B.Q. (2013). Recombinant ADAMTS13 Reduces Tissue Plasminogen Activator-Induced Hemorrhage after Stroke in Mice. Ann. Neurol..

[66] Narasimhan P., Liu J., Song Y.S., Massengale J.L., Chan P.H. (2009). VEGF Stimulates the ERK 1/2 Signaling Pathway and Apoptosis in Cerebral Endothelial Cells After Ischemic Conditions. Stroke J. Cereb. Circ..

[67] Yang C.Q., Li W., Li S.Q., Li J., Li Y.W., Kong S.X., Liu R.M., Wang S.M., Lv W.M. (2014). MCP-1 Stimulates MMP-9 Expression via ERK 1/2 and P38 MAPK Signaling Pathways in Human Aortic Smooth Muscle Cells. Cell. Physiol. Biochem..

[68] Yu S.M., Kim S.J. (2016). Salinomycin Causes Migration and Invasion of Human Fibrosarcoma Cells by Inducing MMP-2 Expression via PI3-Kinase, ERK-1/2 and P38 Kinase Pathways. Int. J. Oncol..

[69] Ismael S., Nasoohi S., Yoo A., Ahmed H.A., Ishrat T. (2020). Tissue Plasminogen Activator Promotes TXNIP-NLRP3 Inflammasome Activation after Hyperglycemic Stroke in Mice. Mol. Neurobiol..

[70] Tang G., Liu Y., Zhang Z., Lu Y., Wang Y., Huang J., Li Y., Chen X., Gu X., Wang Y. (2014). Mesenchymal Stem Cells Maintain Blood-Brain Barrier Integrity by Inhibiting Aquaporin-4 Upregulation after Cerebral Ischemia. Stem Cells.

[71] Anfray A., Drieu A., Hingot V., Hommet Y., Yetim M., Rubio M., Deffieux T., Tanter M., Orset C., Vivien D. (2020). Circulating TPA Contributes to Neurovascular Coupling by a Mechanism Involving the Endothelial NMDA Receptors. J. Cereb. Blood Flow Metab..

[72] Correa F., Gauberti M., Parcq J., Macrez R., Hommet Y., Obiang P., Hernangómez M., Montagne A., Liot G., Guaza C. (2011). Tissue Plasminogen Activator Prevents White Matter Damage Following Stroke. J. Exp. Med..

[73] Kuiper J., Otter M., Rijken D.C., Van Berkel T.J.C. (1988). Characterization of the Interaction in Vivo of Tissue-Type Plasminogen Activator with Liver Cells. J. Biol. Chem..

[74] White S., Lin L., Hu K. (2020). NF-ΚB and TPA Signaling in Kidney and Other Diseases. Cells.

[75] Etique N., Verzeaux L., Dedieu S., Emonard H. (2013). Lrp-1: A Checkpoint for the Extracellular Matrix Proteolysis. BioMed Res. Int..

[76] Kim S.Y., Cheon S.Y., Kim E.J., Lee J.H., Kam E.H., Kim J.M., Park M., Koo B.N. (2017). Isoflurane Postconditioning Inhibits TPA-Induced Matrix Metalloproteinases Activation After Hypoxic Injury via Low-Density Lipoprotein Receptor-Related Protein and Extracellular Signal-Regulated Kinase Pathway. Neurochem. Res..

[77] Lapchak P.A., Han M.K. (2010). Simvastatin Improves Clinical Scores in a Rabbit Multiple Infarct Ischemic Stroke Model: Synergism with a ROCK Inhibitor but Not the Thrombolytic Tissue Plasminogen Activator. Brain Res..

[78] Elshiaty M., Schindler H., Christopoulos P. (2021). Principles and Current Clinical Landscape of Multispecific Antibodies against Cancer. Int. J. Mol. Sci..

[79] Rikitake Y., Kim H.H., Huang Z., Seto M., Yano K., Asano T., Moskowitz M.A., Liao J.K. (2005). Inhibition of Rho Kinase (ROCK) Leads to Increased Cerebral Blood Flow and Stroke Protection. Stroke.

[80] Turner N.A., O’Regan D.J., Ball S.G., Porter K.E. (2005). Simvastatin Inhibits MMP-9 Secretion from Human Saphenous Vein Smooth Muscle Cells by Inhibiting the RhoA/ROCK Pathway and Reducing MMP-9 MRNA Levels. FASEB J..

[81] Kim J.S. (2019). TPA Helpers in the Treatment of Acute Ischemic Stroke: Are They Ready for Clinical Use?. J. Stroke.

[82] Paemen L., Martens E., Norga K., Masure S., Roets E., Hoogmartens J., Opdenakker G. (1996). The Gelatinase Inhibitory Activity of Tetracyclines and Chemically Modified Tetracycline Analogues as Measured by a Novel Microtiter Assay for Inhibitors. Biochem. Pharmacol..

[83] Golub L.M., Ramamurthy N., McNamara T.F., Gomes B., Wolff M., Casino A., Kapoor A., Zambon J., Ciancio S., Schneir M. (1984). Tetracyclines Inhibit Tissue Collagenase Activity. A New Mechanism in the Treatment of Periodontal Disease. J. Periodontal Res..

[84] Murata Y., Rosell A., Scannevin R.H., Rhodes K.J., Wang X., Lo E.H. (2008). Extension of the Thrombolytic Time Window with Minocycline in Experimental Stroke. Stroke.

[85] Fagan S.C., Waller J.L., Nichols F.T., Edwards D.J., Pettigrew L.C., Clark W.M., Hall C.E., Switzer J.A., Ergul A., Hess D.C. (2010). Minocycline to Improve Neurologic Outcome in Stroke (MINOS): A Dose-Finding Study. Stroke.

[86] Noh S.J., Lee S.H., Shin K.Y., Lee C.K., Cho I.H., Kim H.S., Suh Y.H. (2011). SP-8203 Reduces Oxidative Stress via SOD Activity and Behavioral Deficit in Cerebral Ischemia. Pharmacol. Biochem. Behav..

[87] Noh S.J., Lee J.M., Lee K.S., Hong H.S., Lee C.K., Cho I.H., Kim H.S., Suh Y.H. (2011). SP-8203 Shows Neuroprotective Effects and Improves Cognitive Impairment in Ischemic Brain Injury through NMDA Receptor. Pharmacol. Biochem. Behav..

[88] Song H.Y., Chung J.I., Anthony Jalin A.M., Ju C., Pahk K., Joung C., Lee S., Jin S., Kim B.S., Lee K.S. (2022). The Quinazoline Otaplimastat (SP-8203) Reduces the Hemorrhagic Transformation and Mortality Aggravated after Delayed RtPA-Induced Thrombolysis in Cerebral Ischemia. Int. J. Mol. Sci..

[89] Kim J.S., Lee K.B., Park J.H., Sung S.M., Oh K., Kim E.G., Chang D.I., Hwang Y.H., Lee E.J., Kim W.K. (2020). Safety and Efficacy of Otaplimastat in Patients with Acute Ischemic Stroke Requiring TPA (SAFE-TPA): A Multicenter, Randomized, Double-Blind, Placebo-Controlled Phase 2 Study. Ann. Neurol..

[90] Pervin M., Unno K., Takagaki A., Isemura M., Nakamura Y. (2019). Function of Green Tea Catechins in the Brain: Epigallocatechin Gallate and Its Metabolites. Int. J. Mol. Sci..

[91] Martínez-Coria H., Arrieta-Cruz I., Gutiérrez-Juárez R., López-Valdés H.E. (2023). Anti-Inflammatory Effects of Flavonoids in Common Neurological Disorders Associated with Aging. Int. J. Mol. Sci..

[92] Han J., Wang M., Jing X., Shi H., Ren M., Lou H. (2014). (-)-Epigallocatechin Gallate Protects against Cerebral Ischemia-Induced Oxidative Stress via Nrf2/ARE Signaling. Neurochem. Res..

[93] Lim S.H., Kim H.S., Kim Y.K., Kim T.M., Im S., Chung M.E., Hong B.Y., Ko Y.J., Kim H.W., Lee J.I. (2010). The Functional Effect of Epigallocatechin Gallate on Ischemic Stroke in Rats. Acta Neurobiol. Exp. (Wars).

[94] You Y.P. (2016). Epigallocatechin Gallate Extends the Therapeutic Window of Recombinant Tissue Plasminogen Activator Treatment in Ischemic Rats. J. Stroke Cerebrovasc. Dis..

[95] Wang X.H., You Y.P. (2017). Epigallocatechin Gallate Extends Therapeutic Window of Recombinant Tissue Plasminogen Activator Treatment for Brain Ischemic Stroke: A Randomized Double-Blind and Placebo-Controlled Trial. Clin. Neuropharmacol..

[96] Borlongan C.V., Lind J.G., Dillon-Carter O., Yu G., Hadman M., Cheng C., Carroll J., Hess D.C. (2004). Bone Marrow Grafts Restore Cerebral Blood Flow and Blood Brain Barrier in Stroke Rats. Brain Res..

[97] Park H.J., Shin J.Y., Kim H.N., Oh S.H., Song S.K., Lee P.H. (2015). Mesenchymal Stem Cells Stabilize the Blood-Brain Barrier through Regulation of Astrocytes. Stem Cell Res. Ther..

[98] Cheng Z., Wang L., Qu M., Liang H., Li W., Li Y., Deng L., Zhang Z., Yang G.Y. (2018). Mesenchymal Stem Cells Attenuate Blood-Brain Barrier Leakage after Cerebral Ischemia in Mice. J. Neuroinflamm..

[99] Do P.T., Wu C.C., Chiang Y.H., Hu C.J., Chen K.Y. (2021). Mesenchymal Stem/Stromal Cell Therapy in Blood-Brain Barrier Preservation Following Ischemia: Molecular Mechanisms and Prospects. Int. J. Mol. Sci..

[100] Ding X., Li Y., Liu Z., Zhang J., Cui Y., Chen X., Chopp M. (2013). The Sonic Hedgehog Pathway Mediates Brain Plasticity and Subsequent Functional Recovery after Bone Marrow Stromal Cell Treatment of Stroke in Mice. J. Cereb. Blood Flow Metab..

[101] Xin H., Li Y., Shen L.H., Liu X., Hozeska-Solgot A., Zhang R.L., Zhang Z.G., Chopp M. (2011). Multipotent Mesenchymal Stromal Cells Increase TPA Expression and Concomitantly Decrease PAI-1 Expression in Astrocytes through the Sonic Hedgehog Signaling Pathway after Stroke (in Vitro Study). J. Cereb. Blood Flow Metab..

[102] Zanotti L., Angioni R., Calì B., Soldani C., Ploia C., Moalli F., Gargesha M., D’Amico G., Elliman S., Tedeschi G. (2016). Mouse Mesenchymal Stem Cells Inhibit High Endothelial Cell Activation and Lymphocyte Homing to Lymph Nodes by Releasing TIMP-1. Leukemia.

[103] Ries C., Egea V., Karow M., Kolb H., Jochum M., Neth P. (2007). MMP-2, MT1-MMP, and TIMP-2 Are Essential for the Invasive Capacity of Human Mesenchymal Stem Cells: Differential Regulation by Inflammatory Cytokines. Blood.

[104] Liang T., Gao W., Zhu L., Ren J., Yao H., Wang K., Shi D. (2019). TIMP-1 Inhibits Proliferation and Osteogenic Differentiation of HBMSCs through Wnt/β-Catenin Signaling. Biosci. Rep..

[105] Manji H.K., Lenox R.H. (1998). Lithium: A Molecular Transducer of Mood-Stabilization in the Treatment of Bipolar Disorder. Neuropsychopharmacology.

[106] Haupt M., Zechmeister B., Bosche B., Lieschke S., Zheng X., Zhang L., Venkataramani V., Jin F., Hein K., Weber M.S. (2020). Lithium Enhances Post-Stroke Blood-Brain Barrier Integrity, Activates the MAPK/ERK1/2 Pathway and Alters Immune Cell Migration in Mice. Neuropharmacology.

[107] Ji Y.B., Gao Q., Tan X.X., Huang X.W., Ma Y.Z., Fang C., Wang S.N., Qiu L.H., Cheng Y.X., Guo F.Y. (2021). Lithium Alleviates Blood-Brain Barrier Breakdown after Cerebral Ischemia and Reperfusion by Upregulating Endothelial Wnt/β-Catenin Signaling in Mice. Neuropharmacology.

[108] Silachev D.N., Gulyaev M.V., Zorova L.D., Khailova L.S., Gubsky L.V., Pirogov Y.A., Plotnikov E.Y., Sukhikh G.T., Zorov D.B. (2015). Magnetic Resonance Spectroscopy of the Ischemic Brain under Lithium Treatment. Link to Mitochondrial Disorders under Stroke. Chem. Biol. Interact..

[109] Silachev D.N., Plotnikov E.Y., Babenko V.A., Savchenko E.S., Zorova L.D., Pevzner I.B., Gulyaev M.V., Pirogov Y.A., Sukhikh G.T., Zorov D.B. (2016). Protection of Neurovascular Unit Cells with Lithium Chloride and Sodium Valproate Prevents Brain Damage in Neonatal Ischemia/Hypoxia. Bull. Exp. Biol. Med..

